# Association of psoriasis with chronic kidney disease and end-stage renal disease: a systematic review and meta-analysis

**DOI:** 10.3389/fmed.2023.1175477

**Published:** 2023-05-12

**Authors:** Xu Jing, Wen Zhuyuan, Chen Aijun, Xiong Jianxia, Huang Kun, Wang Ping

**Affiliations:** ^1^Department of Dermatology, The First Affiliated Hospital of Chongqing Medical University, Chongqing, China; ^2^College of Pediatrics, Chongqing Medical University, Chongqing, China

**Keywords:** psoriasis, chronic kidney disease (CKD), end-stage renal disease (ESRD), renal injury, meta-analysis

## Abstract

**Background and objective:**

Previous studies have shown that patients with psoriasis are at higher risk of developing chronic kidney disease (CKD) and end-stage renal disease (ESRD) compared with general population; however, data on the differences in the occurrence of CKD and ESRD between patients with psoriasis and non-psoriatic controls are limited and inconsistent. The aim of this study was to carry out a comparison of the probability of suffering CKD and ESRD in patients with or without psoriasis by conducting a meta-analysis of cohort studies.

**Methods:**

Cohort studies on PubMed, Web of Science, Embase and Cochrane Library by March, 2023 were searched for. The studies were screened according to pre-established inclusion criteria. Hazard ratios (HRs) and a 95% confidence intervals (CIs) for the renal outcomes among patients with psoriasis were calculated using the random-effect, generic inverse variance method. Subgroup analysis was related to the severity of psoriasis.

**Results:**

A total of seven retrospective cohort studies were included, including 738,104 psoriasis patients and 3,443,438 non-psoriasis subjects, published from 2013 to 2020. Compared to controls without psoriasis, patients with psoriasis had an increased risk of CKD and ESRD, with pooled hazard ratios of 1.65 (95% CI, 1.29–2.12) and 1.37 (95% CI, 1.14–1.64), respectively. Besides, the incidence of CKD and ESRD is positively correlated with the severity of psoriasis.

**Conclusion:**

This study showed that compared to patients without psoriasis, patients with psoriasis, especially those with severe psoriasis, had a significantly increased risk of developing CKD and ESRD. Considering the limitations of this meta-analysis, more high-quality and well-designed studies are needed in the future to validate our findings.

## 1. Introduction

Psoriasis is a common chronic immune-mediated disease that leads to a substantial burden for 125 million persons worldwide (1). The clinical manifestation of psoriasis is characterized by a scaly erythematous rash, and histologically characterized by epithelial hyperplasia with incomplete keratinization and infiltration of various inflammatory cells (such as lymphocytes and neutrophils) in the dermis (2). Most people with psoriasis experience some damage to their quality of life due to this disease, and many feel a substantial negative impact on their psychosocial wellbeing. In fact, the avoidance-oriented coping due to psoriasis is often the greatest source of daily stress in the lives of patients (3).

A growing body of literature has led to recognition that psoriasis is frequently associated with other diseases that extend beyond the skin. Most comorbidity research on psoriasis has focused on links to cardiovascular diseases, cancers, infections and mental health conditions (4, 5). As the understanding of psoriasis grows, the correlations between psoriasis and renal injury have also been continuously reported (6, 7). Several previous studies have found an increased prevalence of microalbuminuria and renal failure in patients with psoriasis (8, 9). In short, psoriasis can affect all organ systems of the body. Therefore, there is a need to better understand the complex pathogenesis of psoriasis and the associated risk factors.

CKD is a syndrome defined as persistent alterations in kidney structure and function due to various causes, and is determined by an estimated glomerular filtration rate (eGFR) < 60 mL/min/1.73 m^2^ or one or more markers of kidney dysfunction including albuminuria, present for >3 months with specific implications for health. End-stage renal disease is defined when GFR is < 15 mL/min/1.73 m^2^ (10). Although the incidence, prevalence, and progression of CKD vary by race and social determinants of health, it affects ~11% of the population, and people with CKD are five to ten times more likely to die prematurely than to progress to end-stage renal disease (11). Health-related quality of life is significantly lower in patients with CKD than in the general population and declines with decreasing GFR, making it imperative to identify the patients at increased risk for CKD and provide appropriate treatment (12).

Interestingly, possible links between psoriasis and CKD/ESRD have also been studied (13–15). A cohort study with 143,883 psoriasis patients showed that psoriasis was associated with the risk of CKD (HR = 1.05) and ESRD (HR = 1.15). Besides, the severity of psoriasis was related to the increased risk of CKD (for mild psoriasis, HR = 0.99; for severe psoriasis, HR = 1.93) and ESRD (for mild psoriasis, HR = 0.98; for severe psoriasis, HR = 4.15) (16). In recent years, a growing body of literature has examined the relationship between psoriasis and CKD and ESRD, reporting an increased risk of CKD and ESRD in patients with psoriasis. Andrea Conti et al. (17) conducted a cohort study of 219 patients with psoriasis, assessed for CKD and eGFR and albuminuria according to their Kidney Disease Improving Global Outcome (KDIGO) stratified risk criteria, they found that the presence of psoriatic arthritis, the duration of psoriasis (≥21 years) and the magnitude of the Psoriasis Area and Severity Index (PASI) score were positively correlated with the ratio of urinary albumin to creatinine. However, in contrast to the relatively established relationship between those comorbidities and psoriasis, the relationship between psoriasis and CKD/ESRD is less clear.

Considering the uncertainty of causality and potential bias from cross-sectional and case–control studies, we aimed to perform a meta-analysis of cohort studies to understand the association between psoriasis and CKD and ESRD. We hypothesized that psoriasis patients may have potential renal injury. Therefore, in this study, we performed meta-analysis to correlate the development of CKD/ESRD in patients with psoriasis or non-psoriatic patients. With this study, we hope to contribute to a better understanding of the relationship between psoriasis and CKD/ESRD and to provide better evidence-based assessments and recommendations for clinicians and patients.

## 2. Methods

### 2.1. Search strategy

A systematic review of the literature in PubMed, Web of Science, Embase and Cochrane Library databases by March, 2023 was performed. The search strategy was specific for each database and includes a combination of the medical subject heading and free-text terms for psoriasis, chronic kidney disease, and end-stage renal disease. Vocabulary and syntax were adapted for each database. References to the included studies and selected review articles were also manually reviewed for other potentially relevant studies. The details of the search strategy are presented in Supplementary Table 1. We planned that if a potentially eligible study has missing data, we would contact the corresponding author.

### 2.2. Inclusion criteria

The eligibility assessment of each study was conducted independently by two investigators (XJ and WZ), both at the title/abstract and full-text levels. Disagreements between the two reviewers regarding study eligibility were resolved through discussions with a third reviewer (WP). Studies are eligible for inclusion in the meta-analysis if they meet the following criteria: (I) the study design must be cohort studies (prospective or retrospective); (II) the study must included a group of patients with psoriasis and a group of control patients without psoriasis and compared the incidence of CKD and/or ESRD in both groups; (III) studies must report relative risk (RR), hazard ratio (HR), or standardized incidence rate (SIR) with 95% confidence intervals (CI) or provide sufficient raw data for the calculation of these ratios. If patients have CKD and/or ESRD at baseline, then this article will be excluded.

### 2.3. Data extraction

The following information was extracted using a standardized data collection form: name of first author, year of publication, country or region of origin, study population, number of cases and controls, and results: HRs (95% CI) of CKD and ESRD. Two independent reviewers (XJ and WZ) extracted data, and confirmed all entries and checked at least twice for completeness and accuracy. Any disagreements were addressed by discussion a third investigator (WP).

### 2.4. Study quality assessment

The risk of bias and quality of the literature was evaluated using the Cochrane Handbook for Systematic Reviews of Interventions (18) in seven main areas: (I) random sequence generation (selection bias); (II) allocation concealment (selection bias); (III) blinding of participants and treatment providers (performance bias); (IV) blinding of outcome assessors (detection bias); (V) incomplete outcome data (attrition bias); (VI) selective reporting (reporting bias); (VII) other biases. Based on the risk of bias assessment criteria, each entry was judged as one of the three levels of bias: low risk of bias, unclear, and high risk of bias.

### 2.5. Statistical analysis

Data were analyzed using Review Manager 5.4 software from the Cochrane Collaboration (London, UK). The risk of outcome was presented as a log HR with a 95% CI and standard error for included studies. Given that different background populations and the methods used to identify cases, comparators, and outcomes of interest may lead to differences between studies, the DerSimonian–Laird random-effects model was chosen over a fixed-effects model. In addition, we assessed the heterogeneity across studies using the I^2^ statistic: a value of I^2^ of 0–25% represents insignificant heterogeneity, more than 25% but ≤ 50% low heterogeneity, more than 50% but ≤ 75% moderate heterogeneity, and more than 75% high heterogeneity (19). *P* < 0.05 was considered statistically significant, except where otherwise specified. In addition, a stratified analysis was performed based on the severity of psoriasis and the respective risk assessment of mild and severe psoriasis.

## 3. Results

### 3.1. Identification of eligible studies

**Figure 1:**
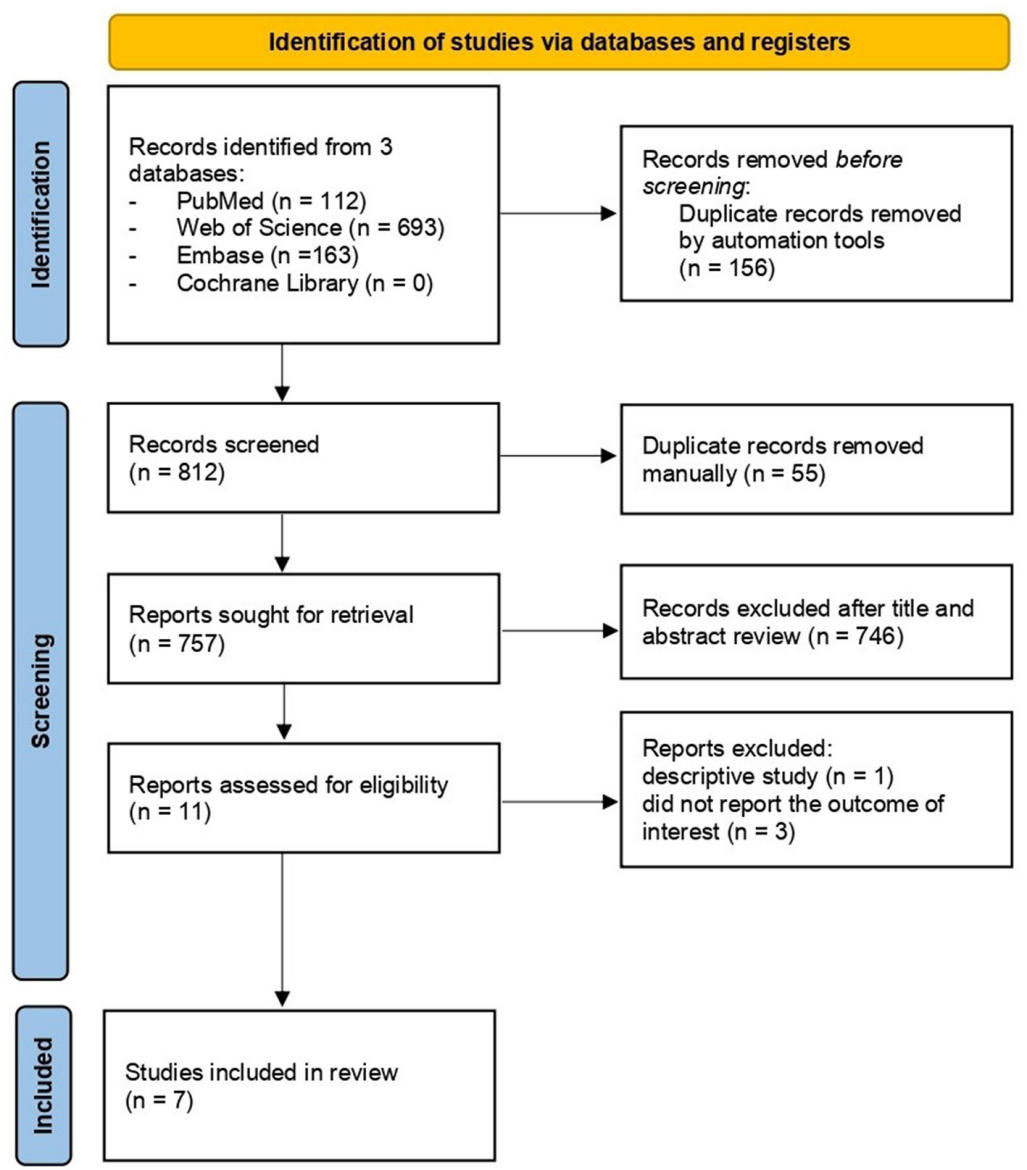
Literature identification and review process.

Figure 1 presents the process of literature search and selection of potential studies. 968 studies were identified, of which 156 duplicates were removed by automation tools; 55 duplicates were removed manually; 746 were excluded after title and abstract reviewing. After reviewing the full texts of the remaining ten articles, four studies were excluded because they did not meet the inclusion criteria: one article was a descriptive study without comparative analysis; the other three articles were excluded since they did not report the outcome of interest (incident CKD and/or ESRD). Finally, seven studies (14–16, 20–23) were included with 738,104 psoriasis cases and 3,443,438 comparators without psoriasis. The mean age of patients with psoriasis was 44.9 years old, compared to 44.1 of the control group without psoriasis. The percentage of male patients with psoriasis was 50.8%, compared to 49.2% in the control group without psoriasis. All the studies were based on managed database studies that relied on diagnosis codes to identify and validate diagnoses of psoriasis, CKD and ESRD. The studies were based on the population databases from the UK, Taiwan and Korea. Adjustment of age and sex was performed for all the seven studies. The characteristics of included studies are shown in Table 1.

**Table 1:** Characteristics of seven studies on association of psoriasis with chronic kidney disease and end-stage renal disease.

**Source**	**Study design and setting**	**Inclusion criteria**	**Study population (No. of participants)**	**HR (95%CI)**	
				**CKD**	**ESRD**
Wan (16) UK	Retrospective cohort THIN	Psoriasis patients aged 18–90	All psoriasis (143,883)	All psoriasis: 1.05 (1.02–1.07)	All psoriasis: 1.15 (0.84–1.58)
			Mild psoriasis (136,529)	Mild psoriasis: 0.99 (0.97–1.02)	Mild psoriasis: 0.98 (0.69–1.38)
			Severe psoriasis (7,354) Comparators (689,702)	Severe psoriasis: 1.93 (1.79–2.08)	Severe psoriasis: 4.15 (1.70–10.11)
Chi (20) Taiwan	Retrospective cohort NHIR	Psoriasis patients	All psoriasis (4,633)	All psoriasis: 1.90 (1.33–2.70)	All psoriasis: 1.14 (0.85–1.53)
			Mild psoriasis (4,180)	Mild psoriasis: 1.01 (0.85–1.19)	Mild psoriasis: 0.90 (0.64–1.29)
			Severe psoriasis (453) Comparators (922,354)	Severe psoriasis: 1.90 (1.33–2.70)	Severe psoriasis: 2.97 (1.72–5.11)
Chiu (21) Taiwan	Retrospective cohort HNIR	Psoriasis patients aged over 18	All psoriasis (4,344)	All psoriasis: 1.47 (1.31–1.64)	All psoriasis: 1.30 (1.01–1.67)
			Mild psoriasis (397)	Mild psoriasis: 1.47 (1.31–1.66)	Mild psoriasis: 1.27 (0.98–1.65)
			Severe psoriasis (3,947) Comparators (13, 032)	Severe psoriasis: 1.49 (1.04–2.14)	Severe psoriasis: 1.62 (0.72–3.64)
Parisi (22) UK	Retrospective cohort CPRD	Psoriasis patients aged over 18	All psoriasis (48,523)	All psoriasis: 1.18 (1.07–1.31)	NA
			Mild psoriasis (46,439)		
			Severe psoriasis (2,084) Comparators (208,187)		
Yu (23) Taiwan	Retrospective cohort NHIR	Psoriasis patients	All psoriasis (3,502) Comparators (10,506)	All psoriasis: 3.00 (2.30–3.93)	All psoriasis: 2.03 (1.04–3.96)
Lee (15) Korea	Retrospective cohort NHIS	psoriasis patients aged over 20	All psoriasis (530,307) Comparators (1,590,921)	NA	All psoriasis: 1.58 (1.47–1.68)
Liu (14) Taiwan	Retrospective cohort NHIR	Patients aged over 20	All psoriasis (2,912) Comparators (8,736)	All psoriasis: 2.48 (1.81–3.40)	NA

CPRD, Clinical practice research datalink; THIN, The health improvement network; NHIR, National health insurance research; NHIS, National health insurance service; CKD, Chronic kidney disease; ESRD, End-stage renal disease; HR, Hazard ratios; CI, Confidence interval; NA, Not available.

### 3.2. Risk of chronic kidney disease among patients with psoriasis

**Figure 2:**
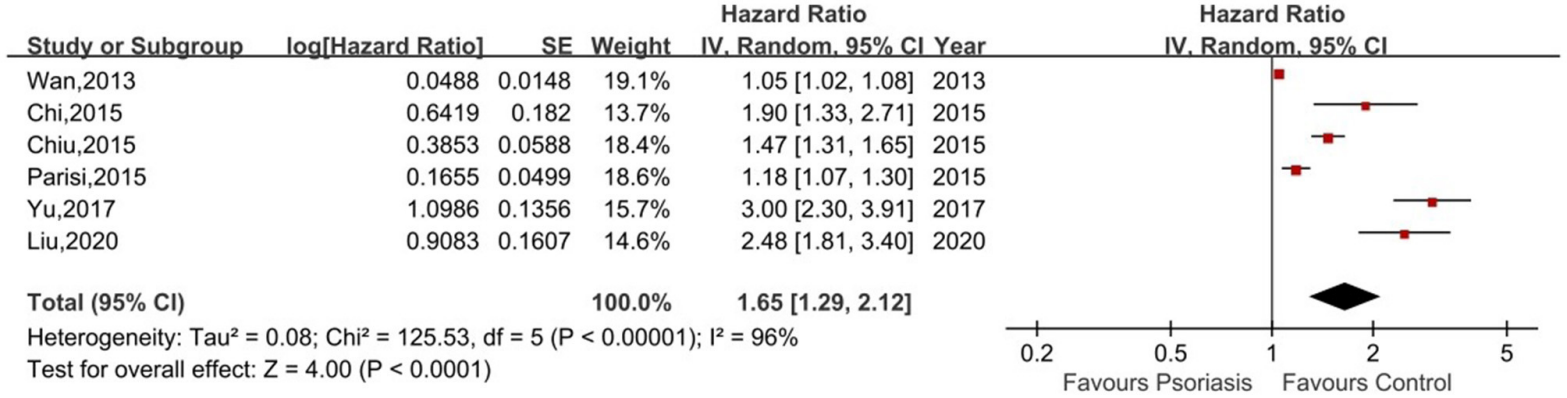
Forest plot of the meta-analysis of risk of chronic kidney disease among patients with psoriasis.

**Figure 3:**
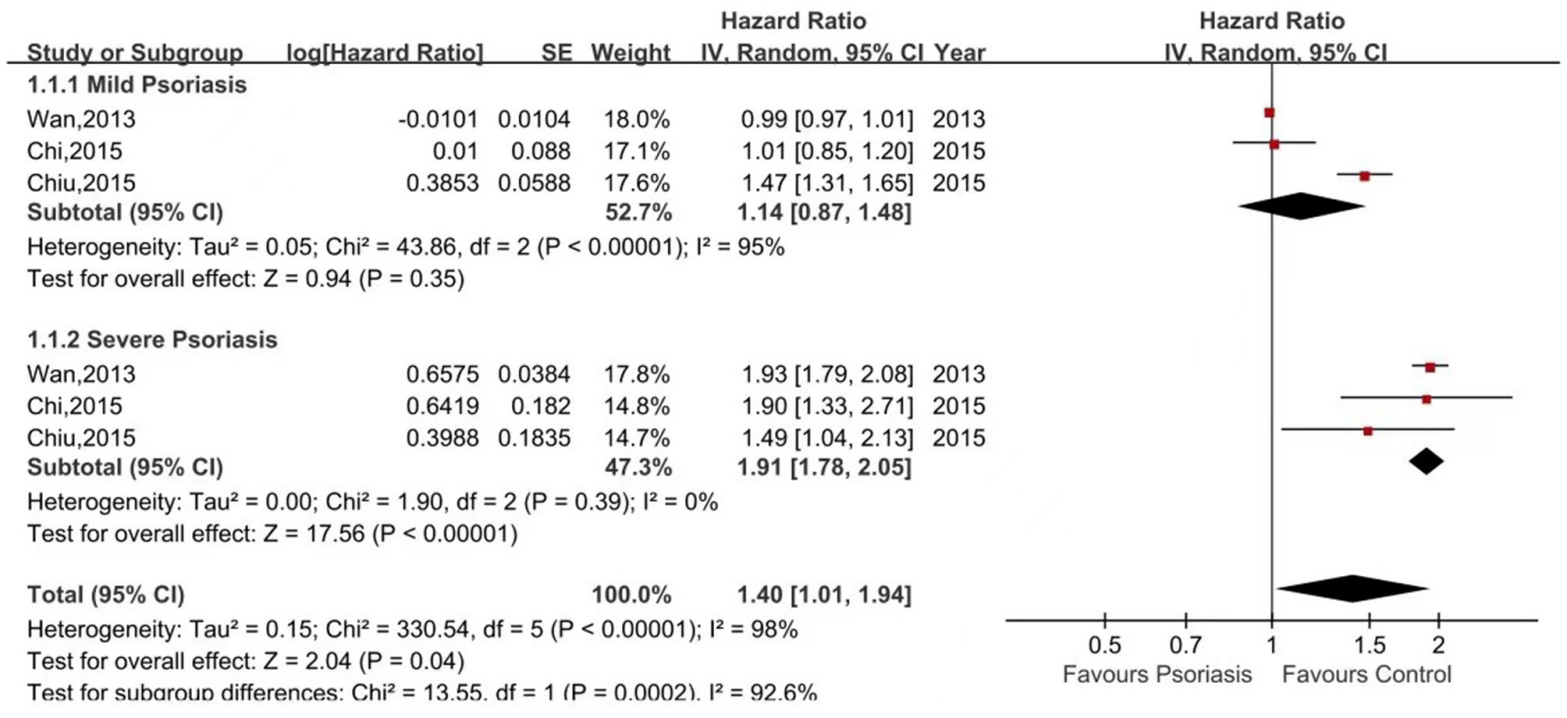
Forest plot of the meta-analysis of risk of chronic kidney disease for patients with mild or severe psoriasis and controls.

Six of the seven studies reported the risk of CKD in psoriasis patients vs. non-psoriasis patients (14, 16, 20–23). Pooled analysis showed a significantly increased risk of CKD in patients with psoriasis, with a pooled HR of 1.65 (95% CI, 1.29–2.12). Statistical heterogeneity was high, with an I^2^ of 96%. A forest plot of the CKD risk meta-analysis is shown in Figure 2. Three studies listed the outcomes of patients with mild and severe psoriasis who developed CKD (16, 20, 21). Figure 3 showed the pool HRs for severe psoriasis and mild psoriasis in subgroup analyses. Meta-analysis showed that patients with severe psoriasis were at higher risk of CKD compared to patients without psoriasis, with a pooled HR of 1.91 (95% CI, 1.78–2.05) and an I^2^ of 0%. However, there was no significant difference in the risk of CKD in patients with mild psoriasis (HR: 1.14; 95% CI: 0.87–1.48) compared to those without psoriasis. The I^2^ value (95%) showed that there was high heterogeneity in the HR for mild psoriasis.

### 3.3. Risk of end-stage renal disease among patients with psoriasis

**Figure 4:**
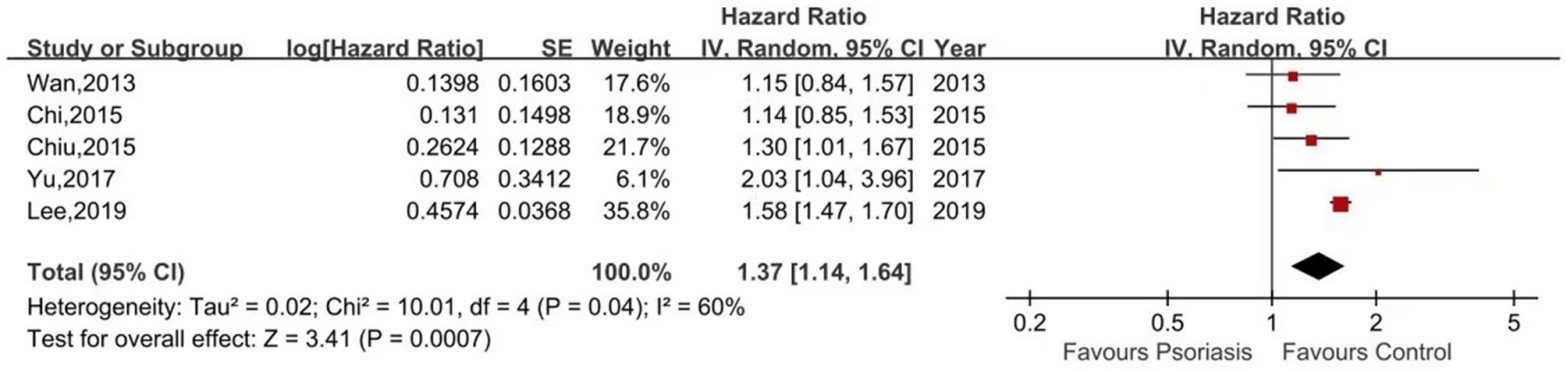
Forest plot of the meta-analysis of risk of end-stage renal disease among patients with psoriasis.

**Figure 5:**
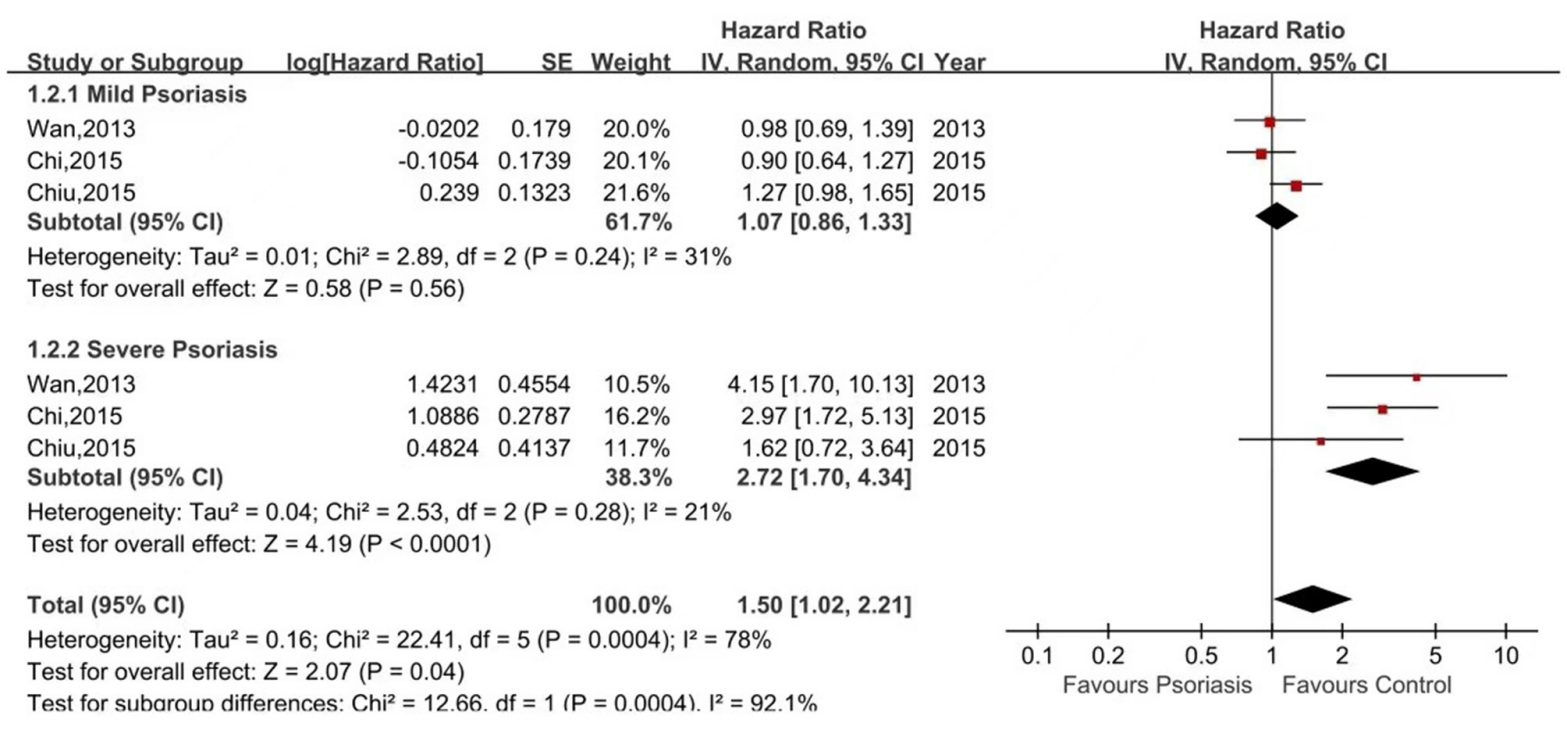
Forest plot of the meta-analysis of risk of end-stage renal disease for patients with mild or severe psoriasis and controls.

Five of the seven studies reported the risk of ESRD in psoriasis patients vs. non-psoriasis patients (15, 16, 20, 21, 23). Pooled analysis showed a significantly increased risk of ESRD in patients with psoriasis, with a pooled HR of 1.37 (95% CI, 1.14–1.64) and an I^2^ of 60%. A forest plot of the CKD risk meta-analysis is shown in Figure 4. Three studies listed the outcomes of patients with mild and severe psoriasis who developed ESRD (16, 20, 21). Figure 5 shows the pool HRs for severe psoriasis and mild psoriasis in subgroup analyses. We found that patients with severe psoriasis had a significantly higher risk of ESRD compared to patients without psoriasis, with a pooled HR of 2.72 (95% CI, 1.70–4.34) and an I^2^ of 21%. However, the risk of ESRD in patients with mild psoriasis (HR: 1.07; 95% CI: 0.86–1.33) was not significantly different from that in patients without psoriasis, with an I^2^ of 31%.

### 3.4. Quality of included studies

**Figure 6:**
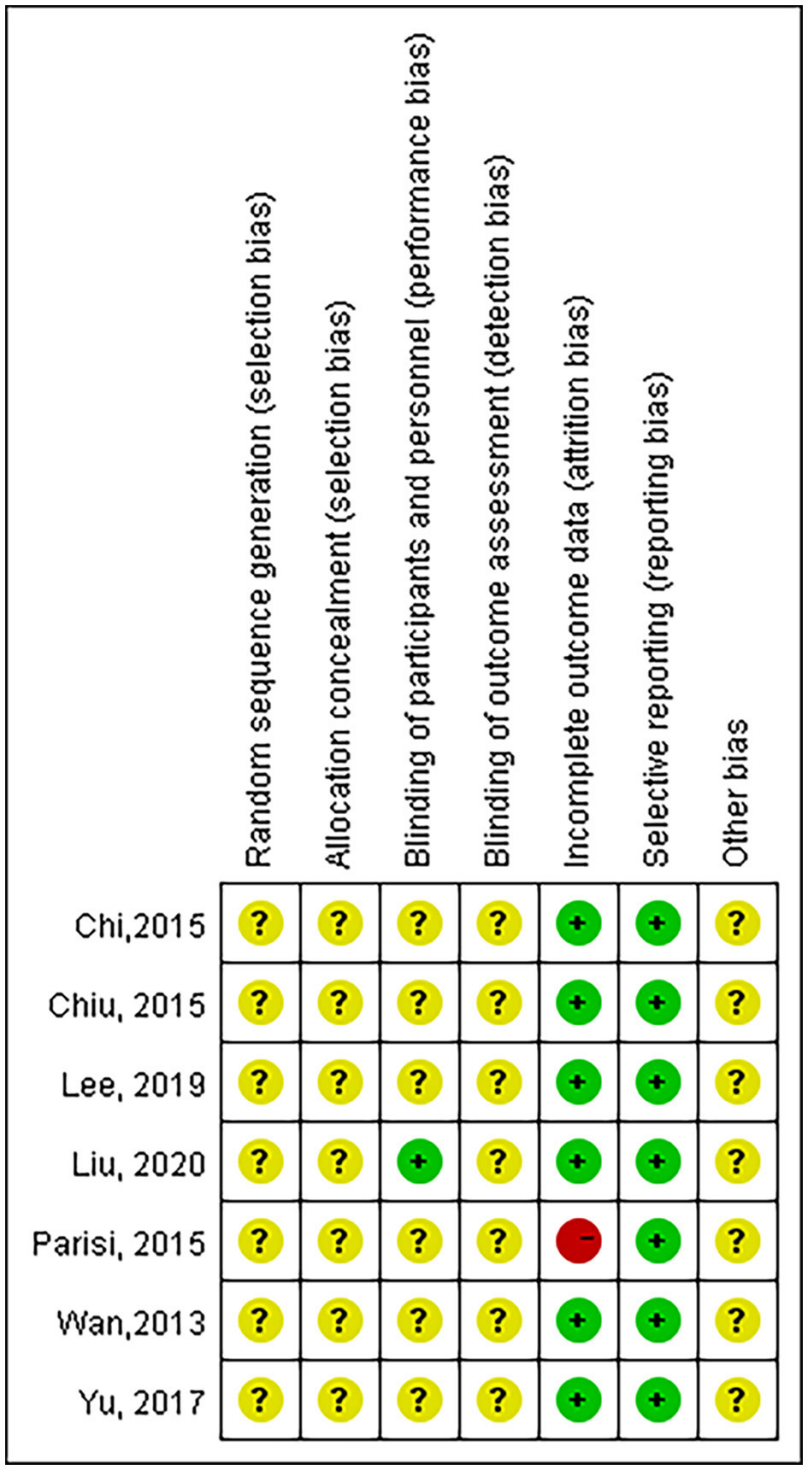
Cochrane Handbook quality assessment scale for cohort studies.

Figure 6 revealed the results of our quality assessment of each of the included articles using the Cochrane Handbook. It can be seen that the included literature only mentioned randomization but did not describe the specific randomization method, and none mentioned allocation concealment; all literature did not mention blinding, except for Liu et al., which mentioned that the researchers analyzed data anonymously under the ambit of personal data protection laws. In terms of outcome data, only Parisi et al. was considered high risk as their study did not adjust its effect estimate for potential confounders. In evaluating the presence of reporting bias, all articles were considered low risk. Because only seven studies were included in our meta-analysis, funnel plots were not used to assess publication bias (18).

## 4. Discussion

We found that patients with psoriasis are at higher risk of incident CKD or ESRD than non-psoriasis patients. In these three articles (16, 20, 21), psoriasis severity was classified as severe or mild based on the use of phototherapy, systemic therapy or not. We found that the risk of CKD and ESRD remained significant in patients with severe psoriasis. However, in patients with mild psoriasis, the risk of ESRD was comparable to that of controls. In addition, the risk of CKD in patients with mild psoriasis remains inconclusive due to the high degree of heterogeneity.

To eliminate potential confounding of metabolic and cardiovascular risk factors in the analysis of correlations between psoriasis and CKD/ESRD, we included only studies that adjusted effect estimates for potential confounders and found that the risk of CKD/ESRD remained significantly higher, suggesting that psoriasis is an independent risk factor for CKD/ESRD. The potential association between psoriasis and CKD/ESRD and the specific underlying pathogenesis of their respective interactions deserve to be explored. Now we discuss the possible mechanisms from inflammation-mediated, drug-related, and chronic renal damages.

Psoriasis is an inflammatory skin disease that histologically manifests itself as epithelial hyperplasia with hyperkeratosis and infiltration of various inflammatory cells in the dermis, suggesting that an inflammatory response manifests the clinical characteristics of psoriasis (24). In addition to inflammation of the skin, associated immune inflammatory cells and inflammatory mediators are also present in the systemic system, and cause inflammatory effects on various organs (25). Among these, over-activation of adaptive immune components is thought to be essential to the pathogenesis of psoriasis and its associated component co-morbidities, such as over-activation of helper T-cell (Th)1 and Th17 lymphocytes, which are important immune cells known to be involved in the pathogenesis of psoriasis (2). It has also been found that Th17 and IL-17 can directly induce renal inflammation by activating neutrophils or by participating in macrophage-mediated tissue damage (26). Chung et al. (27) found significantly higher percentages of Th17 and Th2 in ESRD patients than in healthy controls, as well as significantly higher IL-17 production by effector memory T cells, suggesting that T cell-related immune activation in ESRD patients. In addition, inflammatory factors such as TNF-α and IL-12/23 have important roles in the inflammatory process of psoriasis and are also involved in renal injury. TNF-α can affect renal hemodynamics and renal unit transport, leading to NaCl retention and hypertension (28). IL-23 can drive renal inflammation characterized by massive T-lymphocyte infiltration (29). Veronesi et al. (30) evaluated the renal function of 92 patients with moderate-to-severe plaque psoriasis treated with biologics, including 35 patients with anti-IL-17 biologics, 34 patients with anti-TNF-α biologics and 23 patients with IL-12/23 biologics. Their study found a significant decrease in mean serum creatinine levels after 1 year of treatment from 0.98 ml/min 1 year before to 0.90 ml/min and also a decrease in mean PASI from 17.55 to 1.20, suggesting that treatment targeting psoriasis-causing inflammatory factors improve the underlying kidney damage associated with psoriasis inflammation, and these inflammatory factors may play a role in both psoriasis and kidney damage. In conclusion, both psoriasis and chronic kidney disease involve immune inflammatory mechanisms, but the detailed mechanisms are not yet completely understood.

Drugs used in systemic treatment of psoriasis may cause kidney damage, especially methotrexate and cyclosporine. The etiology of methotrexate-induced kidney injury is related to the precipitation of the drug and its metabolites in the renal tubules and direct damage to renal tubular cells (31, 32). Cyclosporine can cause chronic renal hypoperfusion, and enhance oxidative stress and contributes to tubular apoptosis, tubular atrophy and interstitial fibrosis, leading to chronic kidney injury (33). Although methotrexate and cyclosporine are nephrotoxic, there is no literature suggesting that methotrexate and cyclosporine definitively cause kidney damage in patients with psoriasis. In one of the articles we included (15), psoriasis patients were divided into two subgroups (non-systemically treated group and systemically treated group) to compare the likelihood of ESRD in psoriasis patients and non-psoriasis controls, and it was found that cyclosporin did not impact the risk of ESRD. In addition, vitamin D analogs are reported to benefit the treatment of psoriasis, but some studies point out that potential side effects of oral vitamin D in psoriasis patients include hypercalcemia, hypercalciuria and kidney stones, and long-term overdose of vitamin D intake may lead to bone demineralization (34). In addition to the above traditional oral medications, biologics are also widely used in treatment of psoriasis nowadays, but their safety should be concerned. Maghfour et al. (35) found that biologics do not affect long-term renal function in patients with psoriasis combined with CKD. However, there are few clinical trials investigating the adverse effects of biologics, and most do not exclude the effects of diabetes, psoriatic arthritis, and previous oral medications. Therefore, further research is needed to determine whether biologics are associated with renal injury. We hope that dermatologists will recognize that psoriasis is a systemic disease with its own and related complications that deserve attention, and that therapeutic agents, including traditional oral drugs and biologics, should be carefully considered in order to combat the development of CKD and ESRD in patients with psoriasis, especially in severe cases.

As psoriasis is a systemic disease, patients often have co-morbidities, such as vascular artery plaque formation, insulin resistance and specific tissue and organ damage. Meanwhile, co-morbidities are also interrelated. For example, obesity, hypertension and diabetes are risk factors for coronary heart disease, while obesity is also a risk factor for diabetes and non-alcoholic fatty liver (36–38). Therefore, chronic renal damage due to psoriasis and its co-morbidities may be responsible for the development of CKD/ESRD in patients with psoriasis. Mónica et al. (39) found that patients older than 64 years with a history of hypertension were more likely to develop CKD, and psoriasis could increase the risk of kidney damage in patients with conventional cardiovascular risk factors. Garshick et al. (40) used unbiased whole blood transcriptomic and targeted proteome analysis to find that inflammatory vesicle signaling is a major circulating inflammatory feature in patients with psoriasis and that inflammatory vesicle signaling pathways may contribute to endothelial damage and increased risk of cerebrovascular disease in patients with psoriasis, suggesting that endothelial inflammatory responses and endothelial dysfunction are present in patients with psoriasis. It is interesting to note that endothelial inflammation and dysfunction also play an important role in the pathogenesis of chronic kidney disease. Proteinuria is considered as a subclinical marker of vascular permeability and endothelial damage in diabetic and non-diabetic patients. Increased proteinuria may indicate leakage from glomerular capillaries as a result of vascular endothelial damage (41). An increase of proteinuria in patients with psoriasis suggests a possible correlation between endothelial damage, chronic kidney damage and psoriasis.

This study still has several limitations. First, an obvious limitation of our study is that only three papers discussed the relationship between psoriasis severity and nephropathy, and the raw data may not be sufficient to assess or elucidate the impact of psoriasis severity. Furthermore, the diagnosis of patients with severe and mild psoriasis was based on the use of phototherapy or oral medication and did not include the PASI; therefore, we were unable to accurately assess the causal relationship between the prevalence of CKD/ESRD and the stratification of psoriasis severity. Second, all studies in this meta-analysis used electronic health record databases, and the diagnosis of psoriasis and CKD/ESRD according to the database relied on the diagnosis codes in the database, which may be inaccurate and incomplete to lead to misclassification of effect estimates. Third, the I^2^ test attempts to determine whether there are true differences (heterogeneity) or whether the changes found are consistent with chance only (homogeneity) based on the results of the selected studies. In our findings, CKD statistical heterogeneity was high, with I^2^ values of 96%. High heterogeneity may be the result of different baseline characteristics between cohorts, such as race, gender, and age.

With the increase of the literature on the relationship between psoriasis and the extracutaneous system, there is a growing understanding of psoriasis as an autoimmune disease and systemic inflammatory disorder. However, the risk of CKD and ESRD associated with moderate to severe psoriasis has long been underestimated by all, compared to the psoriasis co-morbidities such as cardiovascular disease and metabolic syndrome. CKD and ESRD have a significant impact not only on the morbidity and quality of life of patients, but also on their mortality. CKD prevention and care has become an important public health policy.

## 5. Conclusion

This meta-analysis may shed light on the burden of advanced kidney injury in patients with psoriasis, suggesting that psoriasis significantly increases the risk of incident CKD/ESRD. Patients with severe psoriasis should be given more attention to because they have a higher likelihood of developing CKD/ESRD. Closer monitoring of renal insufficiency, such as routine screening urine microalbuminuria analysis, serum creatinine and blood urea nitrogen testing, should be considered for patients with moderate to severe psoriasis, which can help with early detection and intervention to reduce the substantial morbidity and mortality associated with chronic kidney disease. Further studies, including well-designed clinical trials, are needed to better understand this association and to provide information about psoriasis. A better understanding of this association provides compelling evidence for clinical practice in the prevention of CKD/ESRD.

## Data availability statement

The original contributions presented in the study are included in the article/Supplementary material, further inquiries can be directed to the corresponding author.

## Author contributions

XJ and WZ: literature search, data extraction, and data analysis. CA and XJ: risk of bias assessment. XJ and HK: software and methods. XJ, WZ, CA, and HK: manuscript drafting and revision. WP: manuscript review and editing and undertook the work of designing this meta-analysis. All authors read and approved the final manuscript.

## References

[1] Griffiths CEM Armstrong AW Gudjonsson JE Barker JNWN. Psoriasis. Lancet. (2021) 397:1301–15. 10.1016/S0140-6736(20)32549-6 33812489

[2] Armstrong AW Read C. Pathophysiology, clinical presentation, and treatment of psoriasis: a review. JAMA. (2020) 323:1945–60. 10.1001/jama.2020.4006 32427307

[3] Matterne U Baumeister SE Apfelbacher CJ. Suicidality and risk of suicidality in psoriasis: a critical appraisal of two systematic reviews and meta-analyses. Br J Dermatol. (2019) 181:717–21. 10.1111/bjd.18108 31074832

[4] Amin M Lee EB Tsai TF Wu JJ. Psoriasis and Co-morbidity. Acta Derm Venereol. 2020;100:adv00033. 10.2340/00015555-3387 31971602 PMC9128942

[5] Bu J Ding R Zhou L Chen X Shen E. Epidemiology of psoriasis and comorbid diseases: a narrative review. Front Immunol. (2022) 13:2484. 10.3389/fimmu.2022.880201 35757712 PMC9226890

[6] Peng J Cao L A. case of idiopathic membranous nephropathy and psoriasis vulgaris. Int Urol Nephrol. (2022) 54:237–8. 10.1007/s11255-021-02851-5 33837903

[7] Grewal SK Wan J Denburg MR Shin DB Takeshita J Gelfand JM. The risk of IgA nephropathy and glomerular disease in patients with psoriasis: a population-based cohort study. Br J Dermatol. (2017) 176:1366–9. 10.1111/bjd.14961 27518038 PMC5303688

[8] Bae EH Kim B Song SH Oh TR Suh SH Choi HS . Proteinuria and psoriasis risk: a nationwide population-based study. J Clin Med. (2021) 10:2356. 10.3390/jcm10112356 34071993 PMC8199156

[9] Dervisoglu E Akturk AS Yildiz K Kiran R Yilmaz A. The spectrum of renal abnormalities in patients with psoriasis. Int Urol Nephrol. (2012) 44:509–14. 10.1007/s11255-011-9966-1 21505751

[10] Charles C Ferris AH. Chronic kidney disease. Prim Care. (2020) 47:585–95. 10.1016/j.pop.2020.08.001 33121630

[11] Webster AC Nagler EV Morton RL Masson P. Chronic kidney disease. Lancet. (2017) 389:1238–52. 10.1016/S0140-6736(16)32064-5 27887750

[12] Collaboration GBDCKD. Global, regional, and national burden of chronic kidney disease, 1990–2017: a systematic analysis for the global burden of disease study 2017. Lancet. (2020) 395:709–33. 32061315 10.1016/S0140-6736(20)30045-3 PMC7049905

[13] Schonmann Y Mansfield KE Mulick A Roberts A Smeeth L Langan SM . Inflammatory skin diseases and the risk of chronic kidney disease: population-based case-control and cohort analyses. Br J Dermatol. (2021) 185:772–80. 10.1111/bjd.20067 33730366 PMC11497311

[14] Liu K-L Tsai W-C Tu H-P Lee C-H. Statin use and the risk of chronic kidney disease in patients with psoriasis: a nationwide cohort study in Taiwan. PLoS ONE. (2020) 15:e0237816. 10.1371/journal.pone.0237816 32841265 PMC7447019

[15] Lee E Han JH Bang CH Yoo SA Do Han K Kim H-N . Risk of end-stage renal disease in psoriatic patients: real-world data from a nationwide population- based cohort study. Sci Rep. (2019) 9:16581. 10.1038/s41598-019-53017-4 31719568 PMC6851155

[16] Wan J Wang S Haynes K Denburg MR Shin DB Gelfand JM. Risk of moderate to advanced kidney disease in patients with psoriasis: population based cohort study. BMJ. (2013) 347:f5961. 10.1136/bmj.f5961 24129480 PMC3805477

[17] Conti A Giovannini L Mandel VD Odorici G Lasagni C Bigi L . Chronic kidney disease in psoriasis: a cohort study. J der deutschen dermatologischen gesellschaft. (2020) 18:438–45. 10.1111/ddg.14087 32311824

[18] Cumpston M Li T Page MJ Chandler J Welch VA Higgins JP . Updated guidance for trusted systematic reviews: a new edition of the cochrane handbook for systematic reviews of interventions. Cochrane Database Syst Rev. (2019) 10:ED000142. 10.1002/14651858.ED000142 31643080 PMC10284251

[19] Higgins JP TS Deeks JJ Altman DG. Measuring inconsistency in meta-analyses. BMJ. (2003) 327:557–60. 10.1136/bmj.327.7414.557 12958120 PMC192859

[20] Chi CC Wang J Chen YF Wang SH Chen FL Tung TH. Risk of incident chronic kidney disease and end-stage renal disease in patients with psoriasis: a nationwide population-based cohort study. J Dermatol Sci. (2015) 78:232–8. 10.1016/j.jdermsci.2015.03.012 25862150

[21] Chiu HY Huang HL Li CH Yin YJ Chen HA Hsu ST . Increased risk of glomerulonephritis and chronic kidney disease in relation to the severity of psoriasis, concomitant medication, and comorbidity: a nationwide population-based cohort study. Br J Dermatol. (2015) 173:146–54. 10.1111/bjd.13599 25511692

[22] Parisi R Rutter MK Lunt M Young HS Symmons DPM Griffiths CEM . Psoriasis and the risk of major cardiovascular events: cohort study using the clinical practice research datalink. J InvestDermatol. (2015) 135:2189–97. 10.1038/jid.2015.87 25742120

[23] Yu S Tu H-P Yu C-L Lee C-H Hong C-H. Is psoriasis an independent risk factor of renal disease? A nationwide retrospective cohort study from 1996 to 2010. Dermatol Sinica. (2017) 35:78–84. 10.1016/j.dsi.2017.02.004

[24] Tashiro T Sawada Y. Psoriasis and systemic inflammatory disorders. Int J Mol Sci. (2022) 23:4457. 10.3390/ijms23084457 35457278 PMC9028262

[25] Korman NJ. Management of psoriasis as a systemic disease: what is the evidence? Br J Dermatol. (2020) 182:840–8. 10.1111/bjd.18245 31225638 PMC7187293

[26] Dolff S Witzke O Wilde B. Th17 cells in renal inflammation and autoimmunity. Autoimmun Rev. (2019) 18:129–36. 10.1016/j.autrev.2018.08.006 30572135

[27] Chung BH Kim KW Sun IO Choi SR Park HS Jeon EJ . Increased interleukin-17 producing effector memory T cells in the end-stage renal disease patients. Immunol Lett. (2012) 141:181–9. 10.1016/j.imlet.2011.10.002 22004873

[28] Mehaffey E Majid DSA. Tumor necrosis factor-α, kidney function, and hypertension. Am J Physiol Renal Physiol. (2017) 313:F1005–F8. 10.1152/ajprenal.00535.2016 28724611 PMC5668589

[29] Li H Tsokos MG Bhargava R Adamopoulos IE Menn-Josephy H Stillman IE . IL-23 reshapes kidney resident cell metabolism and promotes local kidney inflammation. J Clin Invest. (2021) 131:12. 10.1172/JCI142428 33956666 PMC8203450

[30] Veronesi G Guglielmo A Gardini A Sacchelli L Loi C Patrizi A . Biological therapy in patients with psoriasis: what we know about the effects on renal function. Dermatol Ther. (2022) 35:e15202. 10.1111/dth.15202 34773435

[31] Wilsdon TD Whittle SL Thynne TR Mangoni AA. Methotrexate for psoriatic arthritis. Cochrane Database Syst Rev. (2019) 1:CD012722. 10.1002/14651858.CD012722.pub2 30656673 PMC6353064

[32] Yan K Zhang Y Han L Huang Q Zhang Z Fang X . Safety and efficacy of methotrexate for Chinese adults with psoriasis with and without psoriatic arthritis. JAMA Dermatol. (2019) 155:327–34. 10.1001/jamadermatol.2018.5194 30698628 PMC6440263

[33] Elmets CA Leonardi CL Davis DMR Gelfand JM Lichten J Mehta NN . Joint AAD-NPF guidelines of care for the management and treatment of psoriasis with awareness and attention to comorbidities. J Am Acad Dermatol. (2019) 80:1073–113. 10.1016/j.jaad.2018.11.058 30772097

[34] Stanescu AMA Simionescu AA Diaconu CC. Oral Vitamin D therapy in patients with psoriasis. Nutrients. (2021) 13:163. 10.3390/nu13010163 33419149 PMC7825555

[35] Maghfour J Elliott E Gill F Stumpf B Murina A. Effect of biologic drugs on renal function in psoriasis patients with chronic kidney disease. J Am Acad Dermatol. (2020) 82:1249–51. 10.1016/j.jaad.2019.12.043 31881298 PMC7446959

[36] Weber B Merola JF Husni ME Di Carli M Berger JS Garshick MS. Psoriasis and cardiovascular disease: novel mechanisms and evolving therapeutics. Curr Atheroscler Rep. (2021) 23:67. 10.1007/s11883-021-00963-y 34468875 PMC9744099

[37] Gau SY Huang KH Lee CH Kuan YH Tsai TH Lee CY. Bidirectional association between psoriasis and nonalcoholic fatty liver disease: real-world evidence from two longitudinal cohort studies. Front Immunol. (2022) 13:840106. 10.3389/fimmu.2022.840106 35251036 PMC8889012

[38] Wu JJ Kavanaugh A Lebwohl MG Gniadecki R Merola JF. Psoriasis and metabolic syndrome: implications for the management and treatment of psoriasis. J Eur Acad Dermatol Venereol. (2022) 36:797–806. 10.1111/jdv.18044 35238067 PMC9313585

[39] Munera-Campos M Ferrándiz C Mateo L Prior-Español Á Carrascosa JM. Prevalence and stages of chronic kidney disease in psoriasis and psoriatic arthritis: a cross-sectional study. Indian J Dermatol Venereol Leprol. (2021) 87:321. 10.25259/IJDVL_372_19 33769751

[40] Garshick MS Barrett TJ Wechter T Azarchi S Scher JU Neimann A . Inflammasome signaling and impaired vascular health in psoriasis. Arterioscler Thromb Vasc Biol. (2019) 39:787–98. 10.1161/ATVBAHA.118.312246 30760013 PMC6436998

[41] Viazzi F Cappadona F Bonino B Pontremoli R. Chronic kidney disease as a predictor of clinical risk in the elderly. J Geriatr Cardiol. (2016) 13:199–201. 27103913 10.11909/j.issn.1671-5411.2016.03.003 PMC4826888

