# Supplementary material for: Association of psoriasis with chronic kidney disease and end-stage renal disease: a systematic review and meta-analysis
Source: Front Med (Lausanne). 2023 May 12;10:1175477. doi: 10.3389/fmed.2023.1175477 (PMC10213311; doi:10.3389/fmed.2023.1175477)
Supplement: Supplementary file 1 [file Data_Sheet_1.docx]

Supplementary Material

Association of psoriasis with chronic kidney disease and end‑stage renal disease: a systematic review and meta-analysis

XU Jing^1+^, WEN Zhuyuan^1,2+^, CHEN Aijun^1^, XIONG Jianxia^1^, HUANG Kun^1^ and WANG Ping^1*^

^1^ Department of Dermatology, the First Affiliated Hospital of Chongqing Medical University, Chongqing, China

^2^College of Pediatrics, Chongqing Medical University, Chongqing, China

*** Correspondence:** WANG Ping: [wang_ping@hospital.cqmu.edu.cn](mailto:wang_ping@hospital.cqmu.edu.cn)

**Supplementary Table 1.** Search strategy of psoriasis, chronic kidney disease and end‑stage renal disease

| **Database** | **Search Terms** |
| --- | --- |
| PubMed | ((psoriasis[Title/Abstract]) AND (((((((Chronic Kidney Disease[Title/Abstract]) OR (Chronic Renal Disease[Title/Abstract])) OR (Chronic Kidney Insufficiency[Title/Abstract])) OR (Chronic Renal Insufficiency[Title/Abstract])) OR (Chronic Kidney Failure[Title/Abstract])) OR (End-Stage Kidney Disease[Title/Abstract])) OR (ESRD[Title/Abstract]))) AND (("1966/1/1"[Date - Publication] : "2023/3/1"[Date - Publication])) |
| Web of Science | (TS=(psoriasis)) AND TS=(Chronic Kidney Disease* OR Chronic Renal Disease* OR Chronic Kidney Insufficiency* OR Chronic Renal Insufficiency* OR Chronic Kidney Failure* OR End-Stage Kidney Disease* OR ESRD*) AND DOP=(1966-01-01/2023-03-01) |
| Embase | #1 'psoriasis':ti,ab,kw AND [01-01-2016]/sd NOT [02-03-2023]/sd AND [1966-2023]/py  #2 'Chronic Kidney Disease':ti,ab,kw OR 'Chronic Renal Disease':ti,ab,kw OR 'Chronic Kidney Insufficiency':ti,ab,kw OR 'Chronic Renal Insufficiency':ti,ab,kw OR 'Chronic Kidney Failure':ti,ab,kw OR 'End-Stage Kidney Disease':ti,ab,kw OR 'ESRD':ti,ab,kw AND [01-01-2016]/sd NOT [02-03-2023]/sd AND [1966-2023]/py  #3 #1 AND #2 |
| Cochrane Library | #1 (psoriasis):ti,ab,kw with Cochrane Library publication date Between Jan 1966 and Mar 2023, in Trials  #2 (Chronic Kidney Disease):ti,ab,kw OR (Chronic Renal Disease):ti,ab,kw OR (Chronic Kidney Insufficiency):ti,ab,kw OR (Chronic Renal Insufficiency):ti,ab,kw OR (Chronic Kidney Failure):ti,ab,kw OR (End-Stage Kidney Disease):ti,ab,kw OR (ESRD):ti,ab,kw with Cochrane Library publication date Between Jan 1966 and Mar 2023, in Trials  #3 #1 AND #2 |
